# An energy and leakage current monitoring system for abnormality detection in electrical appliances

**DOI:** 10.1038/s41598-022-22508-2

**Published:** 2022-11-02

**Authors:** Md. Morshed Alam, Md. Shahjalal, Md. Habibur Rahman, Himawan Nurcahyanto, Aji Teguh Prihatno, Youngjin Kim, Yeong Min Jang

**Affiliations:** 1 Department of Electronics Engineering, Kookmin University, Seoul, 02707 South Korea; 2 Department of Electrical and Electronic Engineering, University of Liberal Arts Bangladesh, Dhaka, 1207 Bangladesh; 3 Department of Computer Science and Engineering, Pusan National University, Busan, 46241 South Korea; 4 FS Corporation, Daejeon, 34126 South Korea

**Keywords:** Electrical and electronic engineering, Energy infrastructure

## Abstract

Unsafe electrical appliances can be hazardous to humans and can cause electrical fires if not monitored, analyzed, and controlled. The purpose of this study is to monitor the system’s condition, including the electrical properties of the appliances, and to diagnose fault conditions without deploying sensors on individual appliances and analyzing individual sensor data. Using historical data and an acceptable range of normal and leakage currents, we proposed a hybrid model based on multiclass support vector machines (MSVM) integrated with a rule-based classifier (RBC) to determine the changes in leakage currents caused by installed devices at a certain moment. For this, we developed a sensor-based monitoring device with long-range communication to store real-time data in a cloud database. In the modeling process, RBC algorithm is used to diagnose the constructed device fault and overcurrent fault where MSVM is applied for detecting leakage current fault. To conduct an operational field test, the developed device was integrated into some houses. The results demonstrate the effectiveness of the proposed system in terms of electrical safety monitoring and detection. All the collected data were stored in a structured database that could be remotely accessed through the Internet.

## Introduction

In the recent years, incidents of electrical fires have significantly enhanced because of an increasing number of electrical appliances penetrating into electrical distribution systems. In the United States, the third leading cause of fires in homes is cooking and heating equipment, accounting for 10% of the total fire incidents^1^. Over the last few years, electrical fire incidents caused by the failure, malfunction, or degradation of electrical equipment have caused significant casualties and damages. As the insulation of old or damaged appliances wears off, a higher amount of residual current flows through the appliances which generate a massive amount of heat at a particular point that may result in the insulation getting burned. This causes a short-circuit, which is responsible for most fire incidents involving electrical appliances^1^. This hidden danger can be effectively eliminated by quickly detecting the causes of faults in appliances through continuous motoring and warning systems. A residual-current device (RCD) that activates depending on a specified threshold is a common and popular device for determining leakage current. Besides the circuit breaker (CB) function, there are no monitoring systems to detect the condition of malfunctioning appliances.

Load monitoring has entered a new era because of the rapid growth of the IoT and cloud computing technologies^2^. Furthermore, it is a vital technology for assessing appliance usage and consumption, as well as for establishing efficient energy-aware operations and diagnosing any unusual electrical activity in appliances^3^. Intelligent control, review, and alarm for individual appliances could acquire the appliances’ activities easily, thus offering a viable solution for advanced electrical safety monitoring. Therefore, equipment for monitoring and detecting electrical fires has been developed and used as the most effective tools for preventing and managing electrical fires^4^. Moreover, as people have become more conscious of electrical safety issues, there has been a growing need for monitoring the health of particular electrical appliances^3,5^. Hence, the continuous monitoring and analysis of corresponding parameters will be a possible solution to ensure that the equipment is in a safe and serviceable condition.

In^6^, the ZigBee-based energy monitoring system is deployed in renewable energy and smart home systems, where sensor nodes are developed to perform switching applications and measure power parameters. With the integration of WiFi technology, Martani et al.^7^ developed time-series energy consumption monitoring systems by considering human activity and occupancy, and ElShafee et al.^8^ focused on the smart home system.

Their studies did not consider the leakage current monitoring system that is the key parameter for diagnosing an electrical appliance’s health. Furthermore, the authors focused on long-range (LoRa) based data communication systems when considering the monitoring issue in factories^9^, PV systems, and smart cities. However, in most cases, the behavior of electrical appliances is not monitored and diagnosed with leakage current and insulation resistance. For understanding the behaviors of the appliance, different types of load categorized approaches (such as semi-intrusive, intrusive, and non-intrusive approaches) have been applied by considering the corresponding parameters^10–12^. Therefore, the appliance’s leakage current depends on different parameters, such as the applied voltage, insulation, and environmental conditions. In^13^, the appliance’s leakage current properties are analyzed on the basis of the non-intrusive approach, where the device is deployed in the systems without considering communication gateway protocols. In^14^, the time-domain waveform of the leakage current of the different insulator strings depending on the weather condition has been monitored where they have not focused on any particular communication technology for data acquisition. In high-voltage insulators, an alternative approach (i.e., radio service technology) is used to send data using optoelectronic sensors^15^. In^16^, the leakage current monitoring for outdoor insulator and distribution surge arrester has been performed, where the LabVIEWTM platform was used for continuous monitoring and data processing instead of a data server. However, the frequency and time-domain analysis of leakage flux and current are used to develop a non-intrusive approach for identifying and discriminating field winding and damper faults on motor starting time^17^. Moreover, the event detection-based non-intrusive load monitoring methods^3,13^ are applied to identify the casualties of the appliances. In the literature, they used some appliances and corresponding active and reactive power profiles for identifying them. However, it is difficult to follow the same procedure for each type of appliance while the penetration of them is more frequent. Therefore, the scheme is no longer important without considering incoming loads (new) in the applied system. To mitigate the discriminative classifier problem of the leakage current, the proposed state detection algorithm is being used in the system.

After years of improvement, several artificial intelligence methods have been developed. Among these numerous methods, support vector machine (SVM)^18,19^, neural networks^20^ and K-nearest neighbor^21^ have become prominent topics in fault detection. SVM could theoretically analyze with the help of learning theory concepts. The advantage of the SVM over other machine learning techniques is that it minimizes the structural classification risk of the training classifier whereas other techniques perform empirical risk minimization. In addition, it has the potential to handle various large classification problems with large feature spaces and can reach feasible performance in practical problems. However, the proposed system could not deal with the unsupervised learning-based method because of the dynamic electrical appliance characteristic.

The SVM is being applied for detecting the faulty condition of the circuit breakers (CB) based on historic vibration measurement data^22^. Liu et al. proposed a hybrid defect diagnostic model for water quality monitoring devices based on multiclass support vector machines (MSVM)^23^. For diagnosing the faulty condition of three-phase induction motor with an external rotor-bearing system, Gangsar et al. has applied the MSVM algorithm while the features are obtained from the time-domain current and vibration signals^24^. By using features from interharmonic voltages, the MSVM identifies the fault positions within the defective zone^25^. Therefore, Kazemi et al. developed the extended Kalman filter-based SVM model to classify the three-phase residual currents in the primary winding of a transformer, where three residual signals are defined as the discrepancies between the measured and estimated three-phase currents^26^. ESlami et al. adopted SVM for identifying high impedance arcing failures in a distributed generation integrated microgrid where principal component analysis and the Pearson correlation coefficient technique were used to scale down and select features, respectively^27^. The Ref^28^ offers a k-means-based classification algorithm for finding abnormalities in the residual current of a solar system. To identify the residual current defect in low voltage distribution networks, a cooperative training classification model based on an upgraded squirrel search method for a semi-supervised SVM and the k-nearest neighbor is applied in^29^. A protection strategy based on least squares-SVM is designed and developed for residual current and touch current^30^. All aforementioned study deal with SVM based different strategies for fault detection in different systems where the proposed system developed rule-based classifiers for detecting sensor fault and load current fault and MSVM is applied for leakage current fault through proper classification in a household environment. All of the aforementioned studies focus on SVM-based fault detection algorithms for various systems. On the contrary, the proposed system developed rule-based classifiers (RBC) for detecting sensor failure and load current fault, while MSVM is used for leakage current fault in a household environment through proper classification.

**Figure 1:**
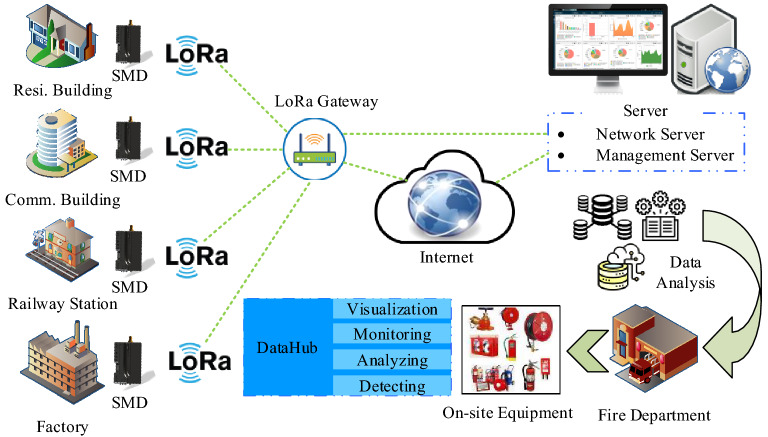
Architecture of the proposed system.

In this study, we propose a fault detection and monitoring system for electrical appliances based on RBC and MSVM. We design and build a microcontroller-based LoRa-sensor-node for data acquisition because of the low power consumption and long-range features of LoRa-based communication networks. We also integrate an AC-DC buck converter to supply power to the sensor. Following that, the system’s real-time fault is detected by RBC-MSVM model. Moreover, this is the first attempt to integrate RBC and MSVM for electrical system fault detection, which contributes to the advancement of monitoring systems in electrical appliances. Unlike in previous studies, the monitoring systems are no longer limited, specifically, in many electrical appliances. Since electrical characteristics may be easily interpreted, this cloud and classification-based continuous monitoring approach is preferred in many electrical systems. Unlike other existing safety devices, such as RCDs, miniature circuit breaker, and molded case circuit breaker, this will ensure the electrical system’s hazard-free operation. In contrast, the proposed system detects leakage current faults by classifying and differentiating them based on correlation and permissible limits acquired from a large amount of historical data in the corresponding system. The permissible range differed according to the system’s conditions; hence, the proposed scheme recognizance this issue because of higher precision.

The following are the advantages of using the proposed framework: All the possible electrical parameters can be known using a single device. Long-range communication is possible because of the deployed LoRa module. Data server will provide essential storage space for handling massive data from a large number of users. Applying the proposed technique for classifying the normal current and the leakage current will help in identifying the causes of fire in the systems. The real-time detection strategy allows to know the system condition before severe damage occurs due to the implementation of multi-class classification. The user may monitor and recognize the present state of the building owing to the accessibility of the web server. The main contributions of our paper are: an integrated safety monitoring device (SMD) based on LoRa is designed and developed by which the electrical parameters can be measured. A sensitivity-based algorithm is implemented for observing and defining the system’s conditions by providing warning specifications. Smooth coordination is enabled through cloud-based control and management architecture for visualization, monitoring, and storing of real-time data. An RBC-MSVM based classifier is used to examine the system’s conditions where the feature selection method has been applied to obtain higher accuracy.

This manuscript is organized as follows: “Methodology” section covers the proposed system’s modeling such as device construction, mathematical modeling, and detection mechanism. “Results and discussion” section contains the simulation findings as well as the explanation that goes along with them. The conclusion of the proposed system is provided in “Conclusion” section.

**Figure 2:**
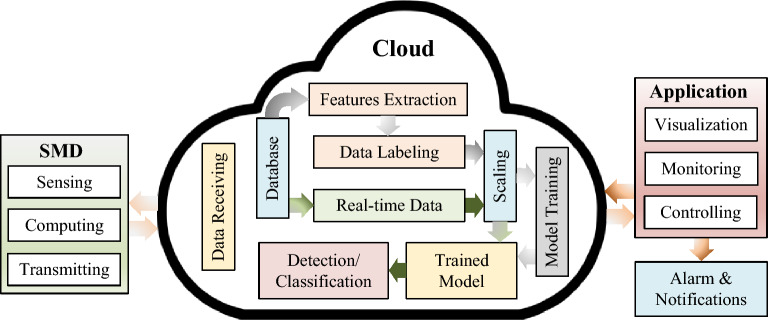
Framework of the proposed methodology.

## Methodology

The proposed system focuses on reducing fires caused by electrical appliances in any location through prompt, dependable monitoring and the use of a control scheme. The proposed system’s framework is depicted in Fig. 1; the process involves collaboration among SMDs, gateway systems, cloud servers, databases, detection algorithms, and visualization. SMDs are used for data acquisition, as shown in Fig. 1, and other necessary features are calculated from the data. Each consumer’s data is transmitted via multiple LoRa gateway channels and uploaded to a cloud server at irregular intervals. The proposed algorithm then categorizes the data based on the acceptable range of leakage current and the number of active appliances. The data from different places are stored and analyzed on the cloud platform because of the increasing number of installed SMDs. Afterward, we applied the proposed RBC-MSVM algorithm to identify the system’s abnormalities.

**Figure 3:**
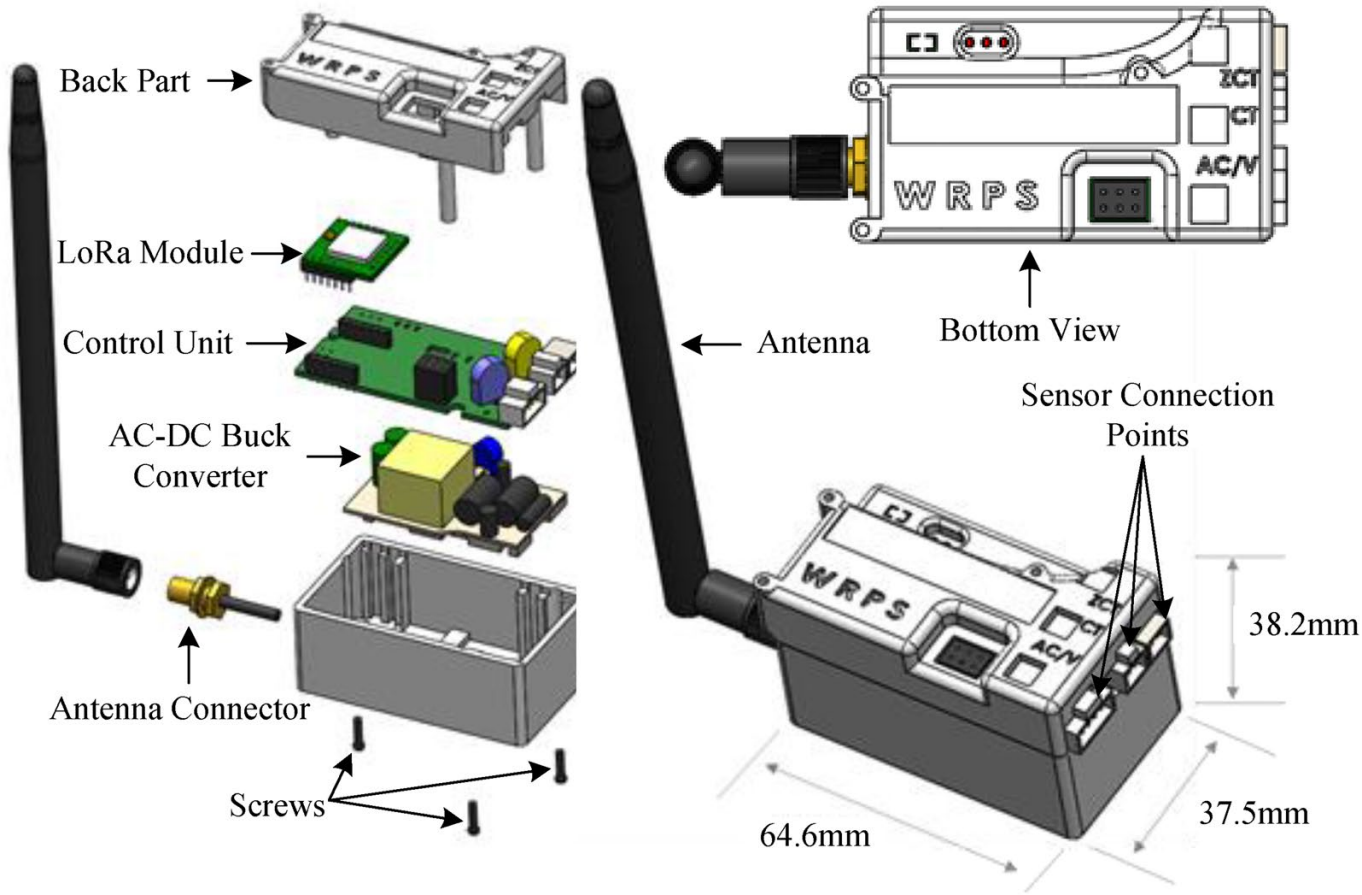
Schematic structure of the safety monitoring device.

Figure 2 illustrates an overview of the proposed methodology, demonstrating the flows of sensing data and information to the cloud database. The system is divided into three parts: the appliance, the database, and the analysis. The appliance section is in charge of acquiring data and transmitting it to the data server via the LoRa module. The database section aims to collect and store sensor data in the database. The relationship between different variables was evaluated in the analysis section to identify the high coloration. Envisaging the households’ appliance specifications, we ascertained the acceptable leakage current to classify the system’s abnormalities. The proposed algorithm will determine the present circumstance regarding the system’s existing issue by investigating the historical data. Furthermore, the analysis section displays the real-time load profiles, leakage current profiles, and the system’s condition. In the following subsection, the detailed methodology is described with other relevant information.

### Device modeling and specification

The schematic diagram of the electrical safety monitoring device is shown in Fig. 3. The device is designed for a single-phase connection rated at 220–380 V (AC) and has the following dimensions: width: 37.5 mm, length: 64.6 mm, and height: 38.2 mm. The LoRa device includes several sensors that measure electrical parameters such as total current, terminal voltage, and leakage currents. From the measured data, we calculated the additional data required for each case, such as total power flow, energy consumption, power factor, resistive and capacitive leakage currents, and insulation resistance. Furthermore, we design in such a way that a multi-step warning signal about the permissible range of total current and residual current concerning the CB’s capacity is provided.

The STM32L microcontroller unit (MCU) handles the overall computation and data indexing. Low-Pass filter and Voltage-Divider are being used in the hardware for better analog data acquisition. Moreover, the STM32L MCU is integrated into the LoRa transceiver device in the proposed system to observe and make a difference in normal conditions. The LoRa system is consisted of end devices, gateways, and a network server that form a star topology with the network server at the root, gateways at level one, and end devices as leaves. The sensed and measured information are accumulated into each LoRa packet. One dedicated channel has been assigned for transmitting the LoRa packet in such an interval that the device remains idle for a certain period in normal operation to reduce power consumption. Furthermore, the device transmits data at very short intervals during the transition from normal to critical conditions. The used LoRa module (SX1276), which is connected to the MCU, sends these data packets to the LoRa gateway module via the 902–928 MHz omnidirectional antenna with a maximum gain of 2dBi. The LoRa network operates in the sub-GHz industrial, scientific, and medical band with maximum transmit powers of 21.7 dBm and 14 dBm in the USA and Europe, respectively^31^. The LoRa modulation (proprietary chirp spread spectrum modulation) uses different types of physical layer packets with different lengths in time, parameterized by the so-called spreading factor (SF), which can take values ${SF \in \mathbb {Z} | 7 \le SF \le 12}.$ The LoRa gateway is used to detect the fault location over a thousand meters because of its proprietary large area coverage^32^. The SF depends on the communication range’s requirement, where the low value of SF means low coverage and vice versa. To store the transmitted data, the interface between the LoRa gateway and the network server is provided by cellular Internet protocol that uses the standard transmission control protocol (TCP).

**Figure 4:**
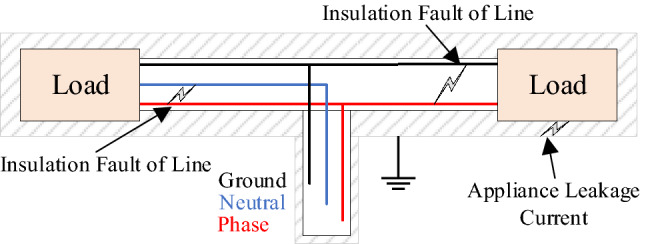
Situation for excessive leakage current.

**Figure 5:**
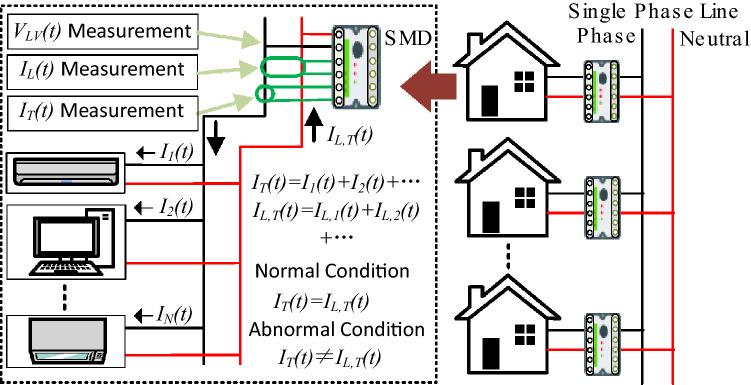
Workflow of the proposed safety monitoring device.

### Mathematical formulation

Figure 4 shows each possible approach of excessive leakage current flow. We demonstrated three scenarios: an insulation fault between the line and the ground, an insulation fault between the line and the neutral, and an appliance fault with the ground. However, Fig. 5 depicts the connection diagram and workflow of the proposed constructed device, which is deployed at the entry point of a low voltage power (i.e., 220–380 V) line in an electrical system (i.e., building, factory, and market). We consider the dynamic characteristic of loads in the proposed systems because electrical appliances are either turned on or off based on the consumer’s demand. The total apparent power of the systems can be defined as follows for *N* loads:

1 $$\begin{aligned} S_{T}(t)= \sum _{ap=1}^{\mathbf {N}} \left\{ P_{ap}(t)+jQ_{ap}(t)\right\} , \end{aligned}$$

where $P_{i}$ and $Q_{i}$ present the active and reactive power of the individual appliance. Therefore, the total currents entering into the loads ($I_{T}(t)=I_{1}(t)+I_{2}(t)+ \cdots$) is as follows:

2 $$\begin{aligned} I_{T}(t)&=I_{Zr,in}(t)+jI_{Xlc,in}(t) \end{aligned}$$

3 $$\begin{aligned} I_{Xlc,in}(t)&= I_{Xl,in}(t)- I_{Xc,in}(t), \end{aligned}$$

where $I_{Xl,in}(t)$ and $I_{Xc,in}(t)$ are the inductive and capacitive currents of the practical load, respectively and $I_{Zr,in}(t)= I_{T}(t)\cos \delta _{I,i}$ and $I_{Xlc,in}(t)= I_{T}(t)\sin \delta _{I,in}$ are the resistive and inductive current flowing to the circuit, respectively. The $\delta _{I,in}$ is also known as the power angle at normal conditions. Similarly, the total amount of returning current $I_{L,T}$ of the system can be defined as follows:

4 $$\begin{aligned} I_{L,T}(t)=I_{Zr,ot}(t)+jI_{Xlc,ot}(t), \end{aligned}$$

where $I_{L,T}(t)$ is defined as the total system current returning to the current sensor. $I_{Zr,ot}(t)= I_{L,T}(t)\cos \delta _{I,ot}$ and $I_{Xlc,o}(t)= I_{L,T}(t)\sin \delta _{I,ot}$ are the resistive and inductive current flowing to the circuit, respectively. Let’s consider a scenario of the system which is explained in Fig. 5.

$$\begin{aligned} \begin{array}{ll} I_{T}(t) = I_{L,T}(t); &{}\quad \text { at normal condition}, \\ I_{T}(t) \ne I_{L,T}(t); &{}\quad \text { at leakage current condition}. \end{array} \end{aligned}$$

The total leakage current ($I_{L}$) flowing out of the connected appliance after considering residual current can be formulated as follows:

5 $$\begin{aligned} I_{L}(t)&=I_{T}(t)-I_{L,T}(t) \end{aligned}$$

6 $$\begin{aligned} I_{L}(t)&= I_{rl}(t)+jI_{cl}(t), \end{aligned}$$

where the resistive and capacitive leakage currents are defined as $I_{rl}= I_{L}(t)\cos \delta _{L}$ and $I_{cl}(t)= I_{L}(t)\sin \delta _{L}$, respectively and $\delta _{L}$ is the angle between $I_{rl}$ and $I_{L}(t)$. Therefore, insulation impedance ($Z_{L}$) is equal to the LV bus $(V_{LV})$ voltage divided by the leakage current that flows through the insulation.

7 $$\begin{aligned} Z_{L} = V_{LV}(t)/I_{L}(t). \end{aligned}$$

**Figure 6:**
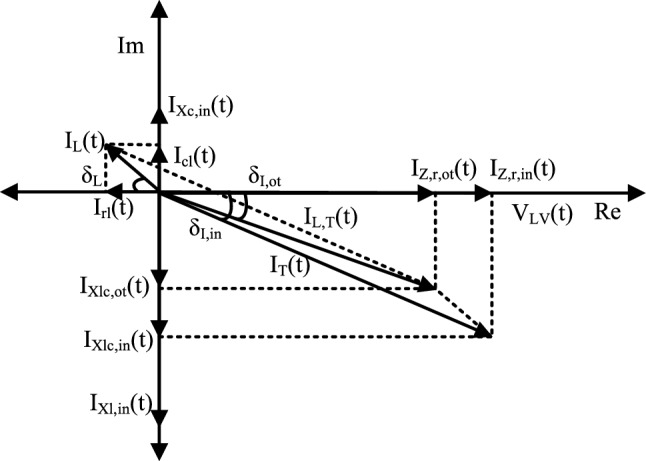
Vector diagram for measuring leakage current.

The quantity of leakage current is quite minimal when compared to the total load current because it only passes via the large insulating impedance of the faulty appliances during the breakdown of insulation. Figure 6 depicts the vector diagram for measuring leakage current wherein the amount of leakage current has considered as large for better visualization. Since the load current is so high in comparison to the $I_L(t)$, the total consumed energy does not differ considerably in normal conditions.

**Figure 7:**
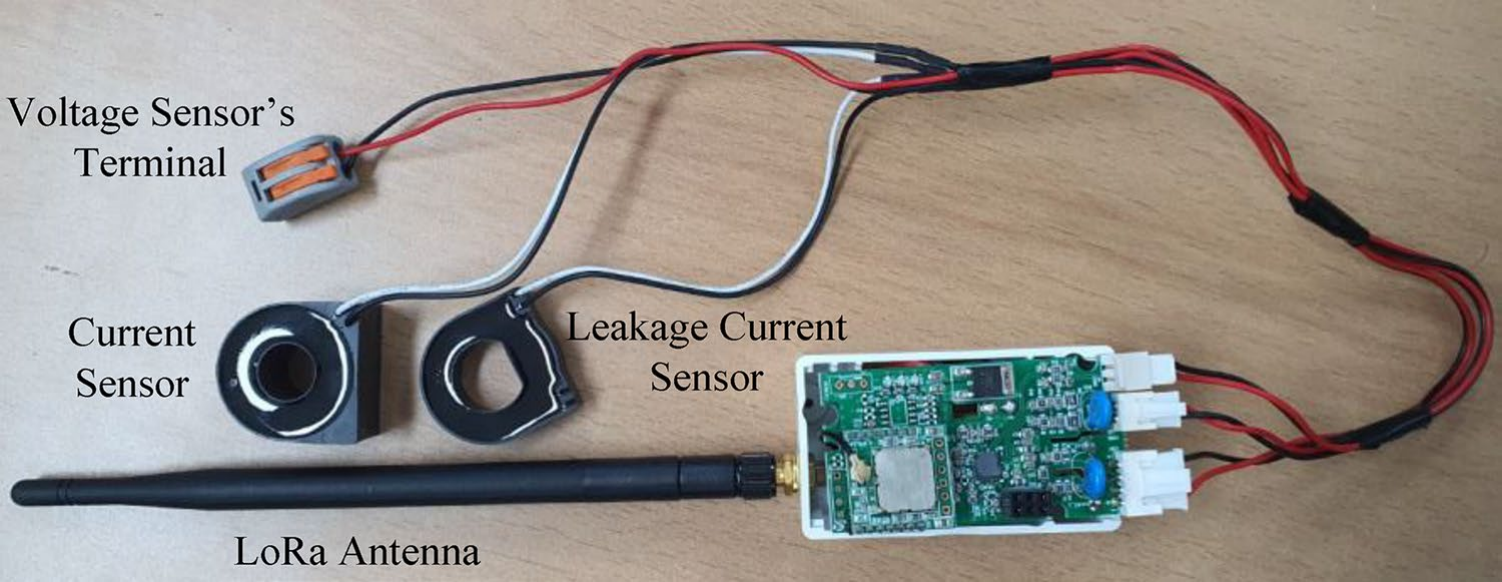
Hardware architecture of the safety monitoring device.

### Data acquisition and classification

Figure 7 shows the SMD device layout. There are two current sensors and one voltage sensor. One current sensor measures the total current of the system and the other sensor measures the leakage current of the system. For measuring the voltage, the terminal of the two wires should be placed as shown in Fig. 5. For measuring the current, the current sensor is only placed on the single wire while both of the wires will be entered inside the leakage current sensor. The leakage current sensor actually measures the difference between the two currents which is described in the Mathematical formulation section. For measuring the phase shift between voltage and current, two operational amplifiers are used for zero-cross detection. Thereafter, both outputs are used as input of an XOR gate. The ON-time of XOR output ( i.e. time difference between two phases) is used to determine the phase shift between voltage and current. Finally, the power factor (p.f.) of the system is measured which is used to determine active and reactive components of the current.

8 $$\begin{aligned} \delta _{I}&= f \times dt_{VI} \times 360, \end{aligned}$$

9 $$\begin{aligned} p.f.&= Cos\delta _{I}, \end{aligned}$$

where *f* and $dt_{VI}$ are defined as frequency and XOR output ON-time, respectively. For measuring the leakage current, we have used a leakage current sensor which is shown in Fig. 7. By using the leakage current and voltage sensor data, the phase angle ($\delta _{L}$) between leakage current and terminal voltage is calculated, similarly. Thereafter, the resistive and capacitive leakage current are measured for the system, accordingly.

10 $$\begin{aligned} \delta _{L} = f \times dt_{VI_{L}} \times 360, \end{aligned}$$

where *f* and $dt_{VI_{L}}$ are defined as frequency and XOR output ON-time, respectively.

However, to ensure greater system security, three warning types are provided. In this case, the over-current protection warning is designed based on the capacity of the deployed CB, whereas a multi-step warning is designed for leakage current protection by differentiating between resistive and capacitive residual currents. The consecutive state of the system *SoS*(*t*) for any consumer is classified by considering the system’s condition.

11 $$\begin{aligned} SoS(t) =\left\{ \begin{array}{ll} SoS^{N};&{}\quad \text {System runs at normal condition} \\ SoS^{W};&{}\quad \text {System runs at warning condition}\\ SoS^{C};&{}\quad \text {System runs at abnormal condition}. \end{array}\right. \end{aligned}$$

In the proposed scheme, we account for the two factors for classifying state and the other two factors for determining the type of appliance. Depending on the different threshold value ranges, the status is defined as $SoS\in \left\{ SoS_{I_{T}},SoS_{I_{L}},SoS_{I_{rl}},SoS_{I_{cl}}\right\}$. The dynamic states of the appliances in terms of total current and leakage currents are defined as $SoS_{I_{T}}\in \left\{ SoS_{I_{T}}^{N},SoS_{I_{T}}^{W},SoS_{I_{T}}^{C}\right\}$, $SoS_{I_{L}}\in \left\{ SoS_{I_{L}}^{N},SoS_{I_{L}}^{W},SoS_{I_{L}}^{C}\right\}$ because of the envisaging three-level warning. For tracing the type of devices, the vulnerability of resistive $SoS_{I_{rl}}\in \left\{ SoS_{I_{rl}}^{N},SoS_{I_{rl}}^{W},SoS_{I_{rl}}^{C}\right\}$ and capacitive leakage currents $SoS_{I_{cl}}\in \left\{ SoS_{I_{cl}}^{N},SoS_{I_{cl}}^{W},SoS_{I_{cl}}^{C}\right\}$ will be taken into consideration. Since the amount of current flow is controlled by the number of contracted appliances and their power rating, the threshold range will be determined accordingly. For additional convenience, we have recommended the opportunity of providing different threshold values. The cut off value of the uninterruptible and healthy system can be defined as $Th_{}^{N}\in \left\{ Th_{I_{T}}^{N},Th_{I_{L}}^{N},Th_{I_{rl}}^{N},Th_{I_{cl}}^{N}\right\}$. In the proposed system, we have considered the intermediate state between the secured and interrupting conditions. The set of range of the interim circumstance of the system is expressed as $Th_{}^{W}\in \left\{ Th_{I_{T}}^{W},Th_{I_{L}}^{W},Th_{I_{rl}}^{W},Th_{I_{cl}}^{W}\right\}$. The excessive current flow causes vulnerable state in the system that is known as critical condition $Th_{}^{C}\in \left\{ Th_{I_{T}}^{C},Th_{I_{L}}^{C},Th_{I_{rl}}^{C},Th_{I_{cl}}^{C}\right\}$. Therefore, the sanctioned constraints of distinguishable apprehension for the $I_{T}$ is as follows:

12 $$\begin{aligned} I_{T,s}^{N}&\le I_{T}(t)\le I_{T,e}^{N},\,\,\,\,\,\, \left\{ I_{T,s}^{N},I_{T,e}^{N} \right\} \in Th_{I_{T}}^{N}, \end{aligned}$$

13 $$\begin{aligned} I_{T,s}^{W}< I_{T}(t)&\le I_{T,e}^{W},\,\,\,\,\,\, \left\{ I_{T,s}^{W},I_{T,e}^{W} \right\} \in Th_{I_{T}}^{W}, \end{aligned}$$

14 $$\begin{aligned} I_{T,s}^{C}< I_{T}(t)&\le I_{T,e}^{C},\,\,\,\,\,\, \left\{ I_{T,s}^{C},I_{T,e}^{C} \right\} \in Th_{I_{T}}^{C}, \end{aligned}$$

where $$\forall I_{T,s}^{N}\approx 0$$, $$\forall I_{T,e}^{N}\approx \forall I_{T,s}^{W}$$ and $$I_{T,e}^{W}\approx \forall I_{T,s}^{C}$$.
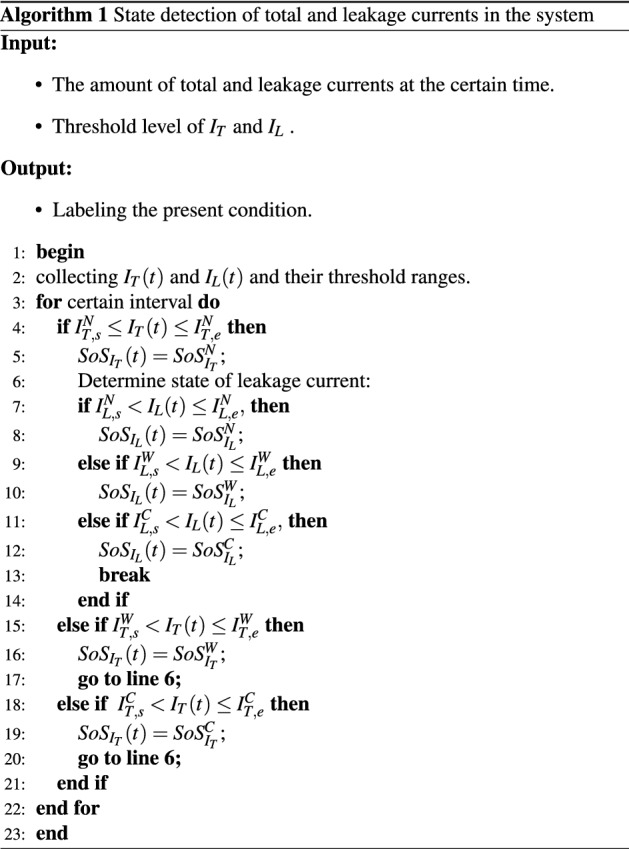


However, the problem associated with leakage current may not remain in the overcurrent flowing system. Consequently, it is mandatory to comprise the leakage current detection to describe whether the system is secured or not. Similarly, the apprehensive state for leakage current will be ascertained based on the following constraints:

15 $$\begin{aligned} I_{L,s}^{N}&\le I_{L}(t)\le I_{L,e}^{N},\,\,\,\,\,\, \left\{ I_{L,s}^{N},I_{L,e}^{N} \right\} \in Th_{I_{L}}^{N}, \end{aligned}$$

16 $$\begin{aligned} I_{L,s}^{W}< I_{L}(t)&\le I_{L,e}^{W},\,\,\,\,\,\, \left\{ I_{L,s}^{W},I_{L,e}^{W} \right\} \in Th_{I_{L}}^{W}, \end{aligned}$$

17 $$\begin{aligned} I_{L,s}^{C}< I_{L}(t)&\le I_{L,e}^{C},\,\,\,\,\,\, \left\{ I_{L,s}^{C},I_{L,e}^{C} \right\} \in Th_{I_{L}}^{C}, \end{aligned}$$

where $\forall I_{L,s}^{N}\approx 0$, $\forall I_{L,e}^{N}\approx \forall I_{L,s}^{W}$ and $I_{L,e}^{W}\approx \forall I_{L,s}^{C}$. The probability of having a leakage issue in multiple devices at the same time is relatively high because of a complete electrical environment inspection. Hence, differentiating resistive and capacitive leakage currents accelerates the process of finding the corresponding appliances. For this reason, we introduced the acceptable range of leakage current using the conditional statement for investigating hazardous circumstances. Furthermore, the permissible limit of the leakage current varies with appliance type, application, and condition. Therefore, the constraints for a reliable and healthy system are defined as follows:

18 $$\begin{aligned} I_{rl,s}^{N}&\le I_{rl}(t)\le I_{rl,e}^{N},\,\,\,\,\,\, \left\{ I_{rl,s}^{N},I_{rl,e}^{N} \right\} \in Th_{I_{rl}}^{N}, \end{aligned}$$

19 $$\begin{aligned} I_{cl,s}^{N}&\le I_{cl}(t)\le I_{cl,e}^{N},\,\,\,\,\,\, \left\{ I_{cl,s}^{N},I_{cl,e}^{N} \right\} \in Th_{I_{cl}}^{N}, \end{aligned}$$

20 $$\begin{aligned} I_{rl,s}^{W}&< I_{rl}(t)\le I_{rl,e}^{W},\,\,\,\,\,\, \left\{ I_{rl,s}^{W},I_{rl,e}^{W} \right\} \in Th_{I_{rl}}^{W}, \end{aligned}$$

21 $$\begin{aligned} I_{cl,s}^{W}&\le I_{cl}(t)<I_{cl,e}^{W},\,\,\,\,\,\, \left\{ I_{cl,s}^{W},I_{cl,e}^{W} \right\} \in Th_{I_{cl}}^{W}, \end{aligned}$$

22 $$\begin{aligned} I_{rl,s}^{C}&< I_{rl}(t)\le I_{rl,e}^{C},\,\,\,\,\,\, \left\{ I_{rl,s}^{C},I_{rl,e}^{C} \right\} \in Th_{I_{rl}}^{C}, \end{aligned}$$

23 $$\begin{aligned} I_{cl,s}^{C}&\le I_{cl}<I_{cl,e}^{C},\,\,\,\,\,\, \left\{ I_{cl,s}^{C},I_{cl,e}^{C} \right\} \in Th_{I_{cl}}^{C}, \end{aligned}$$

where $\left\{ \forall I_{rl,s}^{N},\forall I_{cl,s}^{N} \right\} \in \left[ 0 \right]$, $\forall I_{rl,e}^{N}\approx \forall I_{rl,s}^{W}$, $\forall I_{cl,e}^{N}\approx I\forall _{cl,s}^{W}$ $\forall I_{rl,e}^{W}\approx \forall I_{rl,s}^{C}$, and $\forall I_{cl,e}^{W}\approx \forall I_{cl,s}^{C}$. By applying the given condition in Algorithm 1, we have determined the state of total and leakage currents. Therefore, we have applied Algorithm 2 to identify the current status of resistive leakage in the system. The procedure of finding the capacitive leakage current state is identical to that of determining the resistive leakage current condition; we only provide Algorithm 2 here. Since the boundary of the clusters is very close to each other, the classification algorithm may provide less accuracy. By considering this, we have scaled and re-scaled the features based on the following equations.

24 $$\begin{aligned} E_{k}(C_{k},x_{i})&= (1+C^{2}_{k})*x_{i}, \end{aligned}$$

25 $$\begin{aligned} F_{k}(C_{k},x_{i})&= \frac{E_{k}(C_{k},x_{i})*max(x_{i})}{max(E_{k}(C_{k},x_{i}))}, \end{aligned}$$

where $C_{k}$, $x_{i}$, $F_{k}$ are presented as $k_{th}$ cluster, $i_{th}$ data of the raw feature, and scaled feature which are selected to make up the cluster’s boundary.
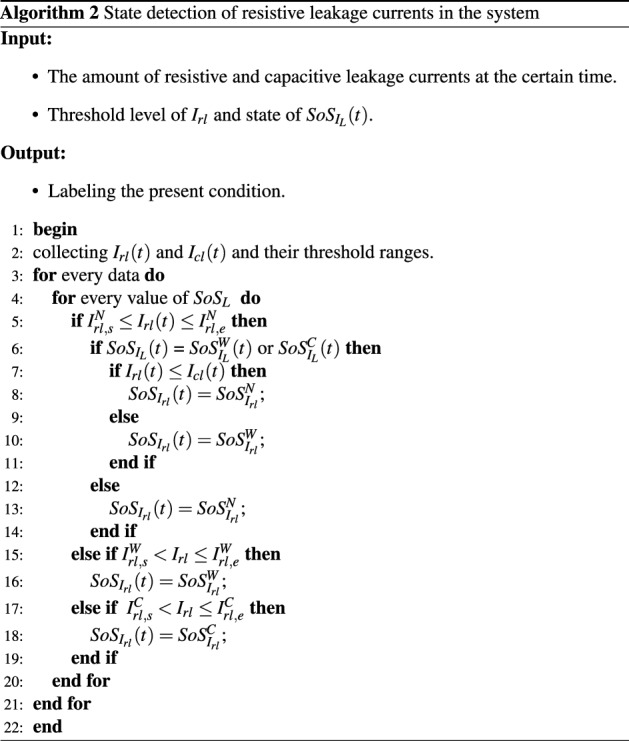


### Database and monitoring

The real-time data storing and monitoring added more value to the electrical safety analysis for understanding the system’s circumstances. Since the leakage current problem and the deterioration of the appliance’s insulation occurred over time, a large amount of data is required to accurately determine the condition of the installed equipment as well as the entire system. As a consequence, the cloud database^33^ is the best option for storing large amounts of data. Cloud computing is a model for providing convenient, on-demand network access to a shared pool of configurable computing resources that can be rapidly provisioned and released with minimal management effort and interaction from service providers. Cloud computing can also help to reduce the administrative burden of program management. The cloud environment enables very diverse data sources to gather information, store it in the cloud database, and feed distinct applications.

In the proposed system, the real-time data packets from the LoRa gateway are sent to the cloud database. On a Windows 10 PC, MySQL version 8.0.19 (Oracle, Co., Austin, TX, USA)^34^ was used as a database management system in the cloud (Microsoft, Redmond, WA, USA). MySQL is a multi-threaded, robust, and scalable open-source service, the platform used under either Oracle’s GNU General Public License or a standard business permit. However, the sensor data collected by the gateway is not uniform and contains noise. Following that, the database server begins intensive computational processing (such as summation, statistics, and data conversion). Finally, the data from several users are stored in the database, which will be used for further processing (such as feature extraction, training, and prediction).

**Figure 8:**
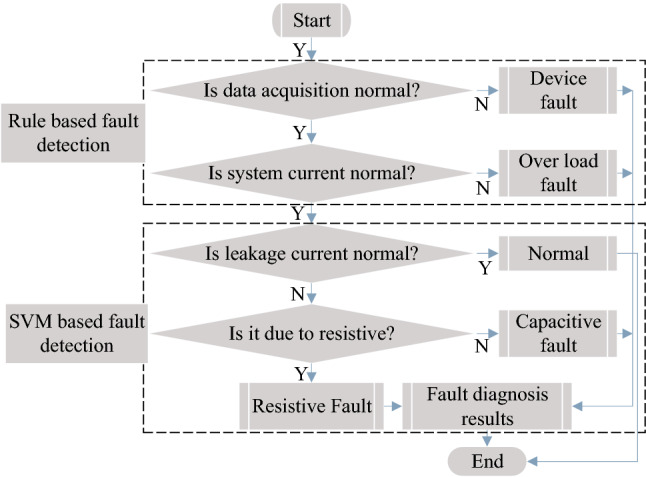
Flow chart of fault diagnosis system.

### Fault classification and detection

In the proposed system, RBC has been applied to determine the device and over-current fault. And MSVM has been used as a discriminative classifier of the system conditions. The flow chart of detecting faults is shown in Fig. 8. In our cases, four rules are generated to diagnose the faults describes as follows:

- Rule1: IF (Sensing data = yes) AND (Current level = normal) THEN the system goes normal
- Rule2: IF (Sensing data = yes) AND (Current level = abnormal) THEN the system goes over-current fault
- Rule3: IF (Sensing data = no) AND (Current level = normal) THEN the system goes device fault
- Rule4: IF (Sensing data = no) AND (Current level = abnormal) THEN the system goes both device and over-current faults

For better classification accuracy, data cleaning, including duplicate and missing data, is conducted prior to categorizing the faulty condition. We have used Pearson’s correlation coefficient-based technique^35^ to remove unnecessary and redundant information and minimize complexity and dimensionality in the proposed system. The density of correlation depends on the Pearson correlation coefficient known as Pearson’s *r*. Let’s consider two variable matrix $S_{T}=[ S_{T_{1}},S_{T_{2}},\cdots , S_{T_{q}}]$ and ${I_{L}=[ I_{L_{1}},I_{L_{2}},\cdots , I_{L_{\mathbf {q}}}]}$, where *q* and $\mathbf {q}$ are represented as samples: $\bar{\gamma _{S_{T}}}=\frac{1}{q}\sum _{a}^{q}S_{T_{a}}$ and $\bar{\gamma _{I_{L}}}=\frac{1}{\mathbf {q}}\sum _{b}^{\mathbf {q}}I_{L_{b}}$. The Pearson correlation co-efficient can be defined as follows:

26 $$\begin{aligned} r_{S_{T},I_{L}}= \frac{\sum _{a=1,b=1}^{q,\mathbf {q}}(S_{T_{a}}- \bar{\gamma }_{S_{T}})(I_{L_{b}}- \bar{\gamma }_{I_{L}})}{\sqrt{\sum _{a=1}^{q}(S_{T_{a}}- \bar{\gamma }_{S_{T}})^2}\sqrt{\sum _{b=1}^{\mathbf {q}}(I_{L_{b}}- \bar{\gamma }_{I_{L}})^2}}. \end{aligned}$$

Similarly, the value of *r* is calculated by taking into account the other variables, with the feature being selected depending on the greater value of *r*.

To classify datasets, it tries to create an optimal hyperplane between two classes of the data set^19^. The hyperplane acts as a decision boundary to categorize the data into different classes. The points nearer to the hyperplane called support vector, are used to determine the optimized hyperplane. For a given training sample $\left\{ (x_i,y_i) \right\} ,\forall i\in \left\{ 1,2,3,....,n \right\}$, where $y_i \in \left\{ +1,-1 \right\}$ represents class labels, optimal hyperplane is determined by the following mathematical expression:

27 $$\begin{aligned} \theta ^{T}x_{i} + b = 0, \end{aligned}$$

where $\theta =\left[ \theta _{1},....,\theta _{n} \right]$ is *n*-dimensional vector of weights and $x_{i}=\left[ x_{1},x_{2},....,x_{n} \right]$ is an *n*-dimensional input vector, and *b* is termed as the biasing unit. Here, *n* represents number of features. The optimization problem associated with finding the hyperplane can be expressed as follows:

28 $$\begin{aligned} min(\theta )\frac{1}{2}\sum _{i=1}^{n}(\theta )^2=\frac{1}{2}\left\| \theta \right\| ^2 =\frac{1}{2}\theta ^{T}\theta , \end{aligned}$$

which is subjected to,

29 $$\begin{aligned} \theta ^{T}x_{i} + b \ge +1&\ \text {if}&\ y_{i}=+1, \end{aligned}$$

30 $$\begin{aligned} \theta ^{T}x_{i} + b \le +1&\ \text {if}&\ y_{i}=-1. \end{aligned}$$

The final nonlinear decision function can be obtained as follows:

31 $$\begin{aligned} f(x)=sign\left( \sum _{i=1}^{n} \alpha _{i} \left( \theta ^{T}x_{i} \right) +b \right) . \end{aligned}$$

To come up with a set of complex features, SVM uses a technique called Kernel $k(x_i,x)$. The value $k(x_i,x)$ corresponds to $\varphi (x_{i}).\varphi (x)$ which maps linearly non-separable patterns into a higher dimension feature space. Finally, the decision function can be modified as follows:

32 $$\begin{aligned} \begin{aligned} f(x)&=sign\left( \sum _{i=1}^{n} \alpha _{i} k(x_{i},x) +b \right) =sign\left( \sum _{i=1}^{n} \alpha _{i} (\varphi (x_{i}).\varphi (x)) +b \right) . \end{aligned} \end{aligned}$$

**Table 1:** Kernel function for the proposed system.

Type of Kernel function	Kernel function
Linear	$x^{T}x_{i}+c$
RBF	$exp\left( \left| -\frac{\left\| x-x_{i} \right\| ^2}{2\sigma ^2} \right| \right)$
Poly	$\left( x^{T}x_{i}+c\right) ^{p}$
Sigmoid	$tanh\left( x^{T}x_{i}+c\right)$

In this study, we have performed the classification experiment taking account into four kernel functions (linear, polynomial, radial basis function (RBF), sigmoid) described in Table 1. Moreover, we have used one versus rest manner multiclass approach. According to this approach, for a *m*th class classification problem *m*th class are trained as positive samples while the rest are treated as negative samples^21,36^.

**Figure 9:**
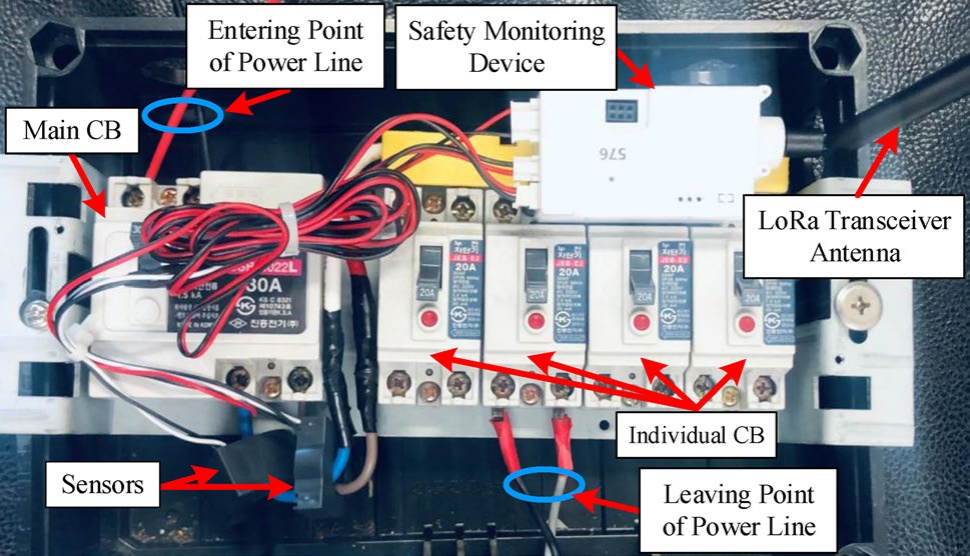
Installation of the SMD with CB.

**Figure 10:**
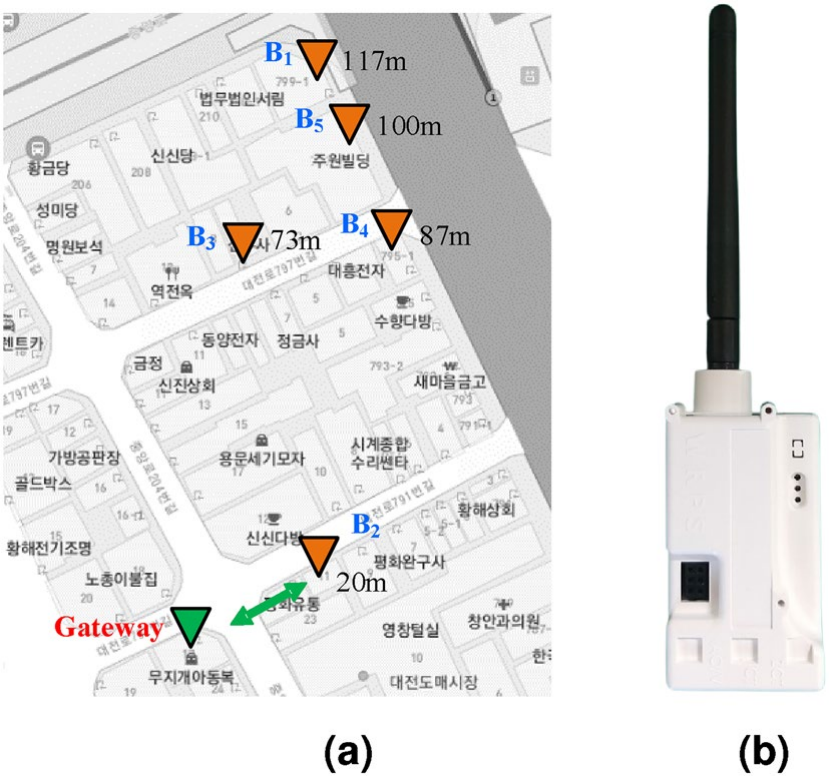
(**a**) Map with nodes’ positions and (**b**) Safety monitoring device.

**Figure 11:**
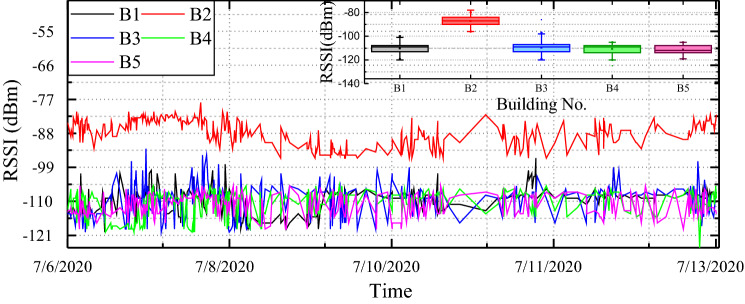
RSSI status of the several LoRa sensor nodes.

**Figure 12:**
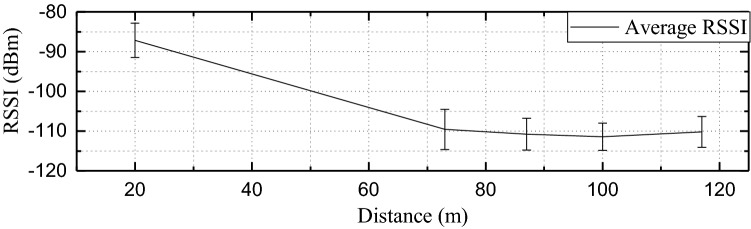
Average RSSI with different distance.

## Results and discussion

The proposed SMD is implemented in a real-world system to ensure its accountability and efficacy. Therefore, we integrated the system in a residential building to evaluate the proposed detection technique, in which the device is placed at the building’s power line entrance point. The installation of SMD is depicted in Fig. 9, with various points noted for interpretation. Two independent current sensors have been installed next to the main CB to detect total and leakage currents. Therefore, single voltage sensor has measured the terminal voltage. During the experiment, we have deployed more than 30 units in Daejon, South Korea. From this, we have selected five buildings that have excessive leakage current. In this experimental study, we have measured the total active power, voltage, current, leakage current (resistive, capacitive, and total), frequency, p.f., and insulation resistance to extract some exigent features for performing classification in turn. The system’s condition is also determined on the basis of the proposed algorithm. The real-time measured data are accumulated into each LoRa packet. The LoRa gateway receives the data and sends it to the server through the Internet by the TCP/Internet protocol. The cloud database enables us to perform real-world verification: monitoring a large number of electrical systems and storing a large amount of data. The study with the proposed system lasted for a few months due to the necessity of a large amount of data.

Moreover, the LoRa transmitting node’s position concerning the GW has been represented in the MAP as shown in Fig. 10a. And Fig. 10b shows the proposed manufactured electrical safety monitoring device. The LoRa received signal strength indicators (RSSIs) for the five different buildings are represented in Fig. 11. The farthest building ($B_{1}$) from the LoRa gateway is located at 117 m distance and experiences RSSI of − 110.18 dBm on average. However, $B_{5}$ has the lowest RSSI value (− 111.4 dBm on average) as it faces a higher amount of attenuation due to the building blockage than $B_{1}$. Among the five buildings $B_{2}$ is the nearest and located 20 m farther from the gateway. It receives LoRa packets with an RSSI value of − 87.17 dBm on average and has the lowest deviation in RSSI values due to almost uniform path loss gain. Figure 12 shows the distribution of average packet RSSI variation with the distance. The sensor nodes located at 73 m and 100 m distances have the highest and lowest variation in RSSI, respectively. Moreover, we have tested the LoRa packet loss rate (PLR) while receiving transmitted packets at the LoRa gateway node. We observed a PLR of 0.5% at the communication distance of 20 m. When we considered a non-line of sight communication at about 120 m the successful packet reception rate was decreased slightly. At 120 m communication distance which is the maximum value in our case, we evaluate a PLR of 2%.

**Figure 13:**
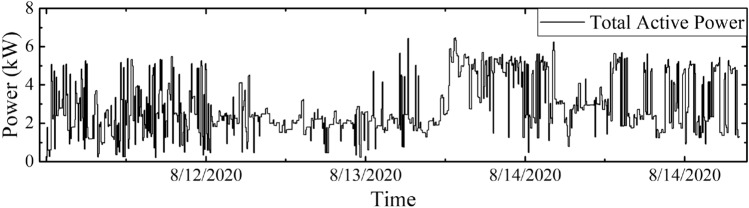
Total power monitoring result in $B_{1}$.

**Figure 14:**
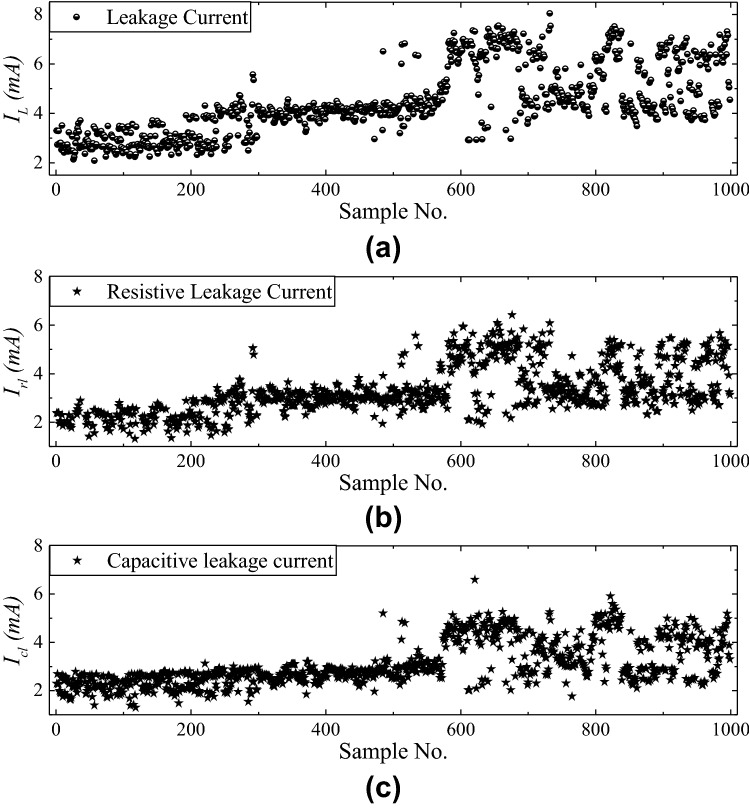
Data sample of (**a**) Total leakage current, (**b**) Resistive leakage current and (**c**) Capacitive leakage current for $B_{1}$.

**Figure 15:**
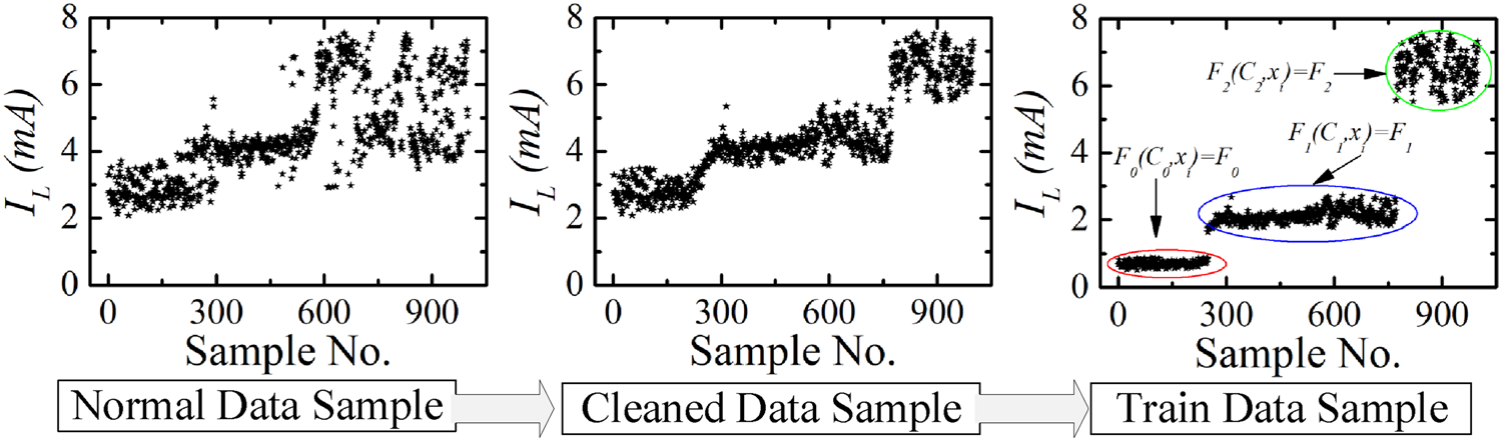
Clustering result of total leakage current for $B_{1}$.

**Figure 16:**
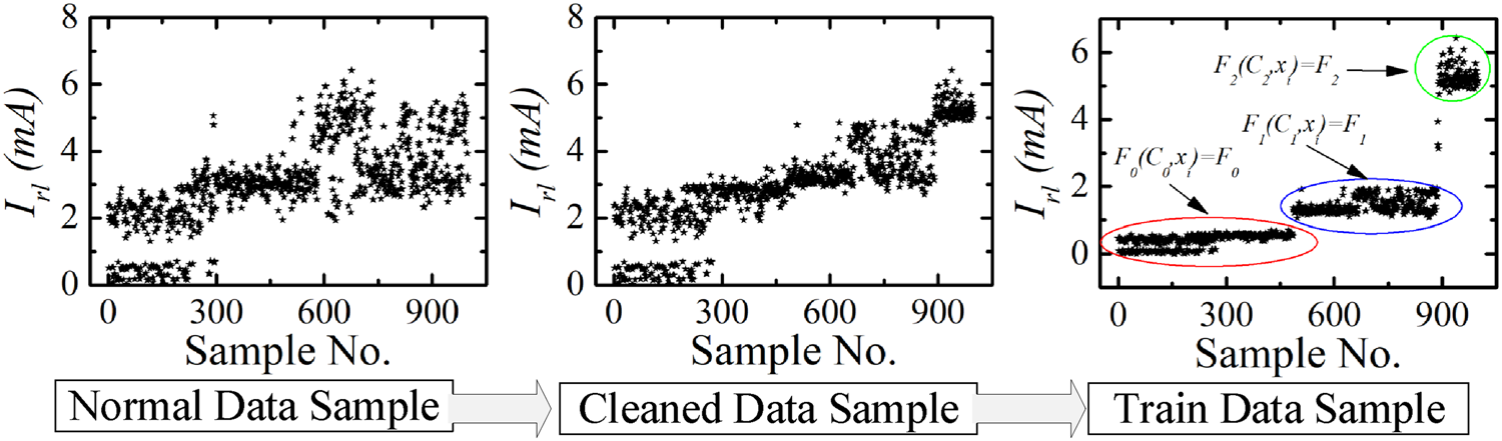
Clustering result of resistive leakage current for $B_{1}$.

**Figure 17:**
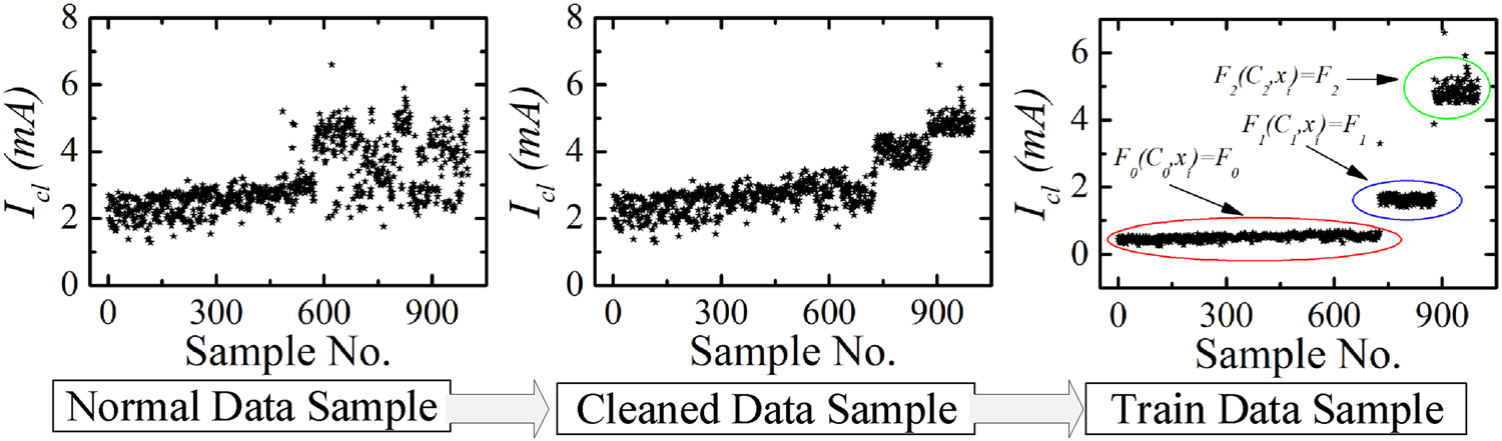
Clustering result of capacitive leakage current for $B_{1}$.

One monitoring sample result of building 1 ($B_{1}$) is shown in Figs. 13 and 14. The total power, total leakage, resistive leakage, and capacitive leakage currents profile are depicted for a few days when the connected load alteration is perceptible. Hence, we showed the data from 0:00 (8/12/2020) to 23:58 (8/18/2020) at an irregular interval from the approximately six-month data. As shown in the figure, the leakage current increases to about 6.5 mA at the moment of the raising of the connected load and restores to normal at the moment of removing the load. However, the proposed algorithm is applied to obtain the optimal decision boundary referred to as the target value. By applying the algorithm, the clustered result of leakage current is illustrated in Figs. 15, 16 and 17, where the three clustered regions are traced with several colors. Hence, we have presented the normal data sample to a trained data sample of leakage currents after cleaning and profiling them. As shown in the figures, the leakage current increases by approximately 8 mA, where the resistive and capacitive leakage currents reach 6.5 and 6.51 mA, respectively. Within the data acquisition time, the event rate of undergoing critical and warning conditions is much lower than that in normal conditions, where the critical moment frequency of the resistive leakage current is higher than that of the capacitive leakage current. We analyzed the relationship between the features by using the features selection technique to select five out of eight.

**Figure 18:**
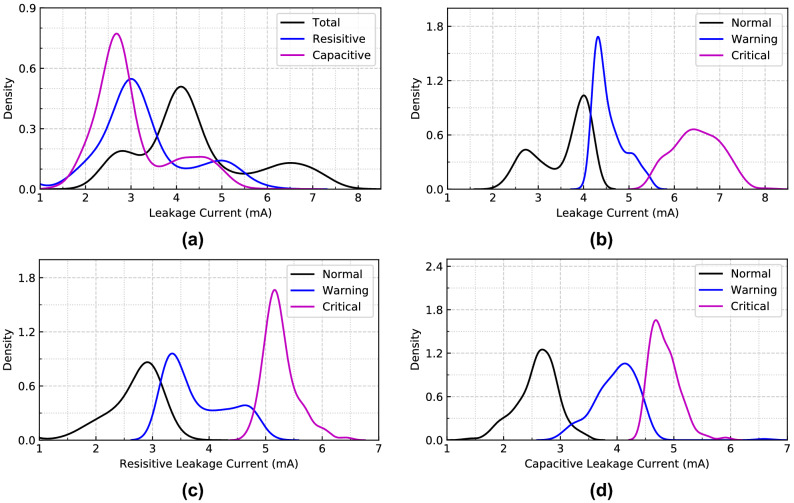
Probability distribution graph of (**a**) Leakage currents (before clustering), (**b**) Total leakage current, (**c**) Resistive leakage current, and (**d**) Capacitive leakage current (after clustering) for $B_{1}$.

Figure 18 presents the continuous probability density of the leakage currents. We aimed to show the data distribution of each clustering range in this figure. In Fig. 18a, the distribution density of total and leakage currents in different observations is presented without considering clustering. From the figure, the flow of leakage current in the middle zone’s range is higher than the other two in this building. Similarly, the clustering range of each class is determined by the permissible limits for total, resistive, and capacitive leakage currents, as illustrated in Fig. 18b–d. Since leakage current warnings can be caused by either the system’s resistive or capacitive load, it can occasionally offer imbalanced data distribution of resistive and capacitive leakage currents.

**Figure 19:**
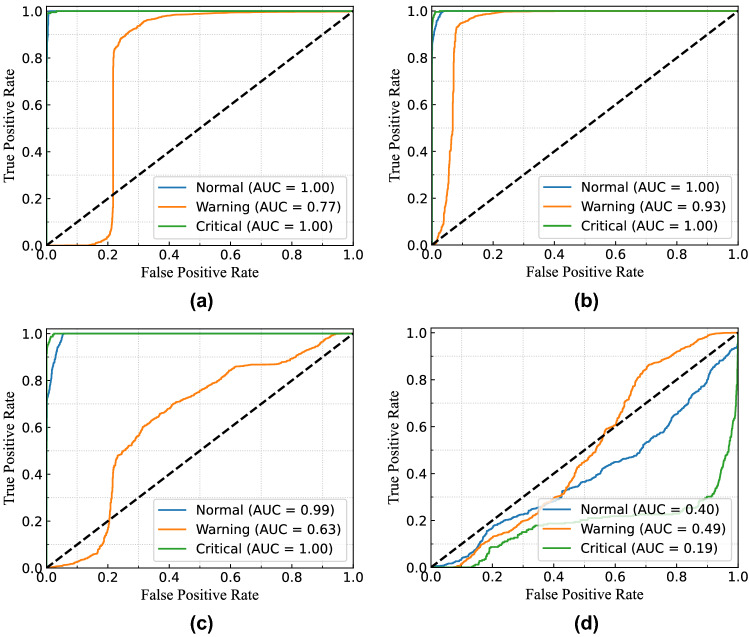
ROC curve of MSVM classifier for total leakage current with (**a**) linear kernel; (**b**) RBF kernel; (**c**) polynomial kernel; and (**d**) sigmoid kernel for $B_{1}$.

**Figure 20:**
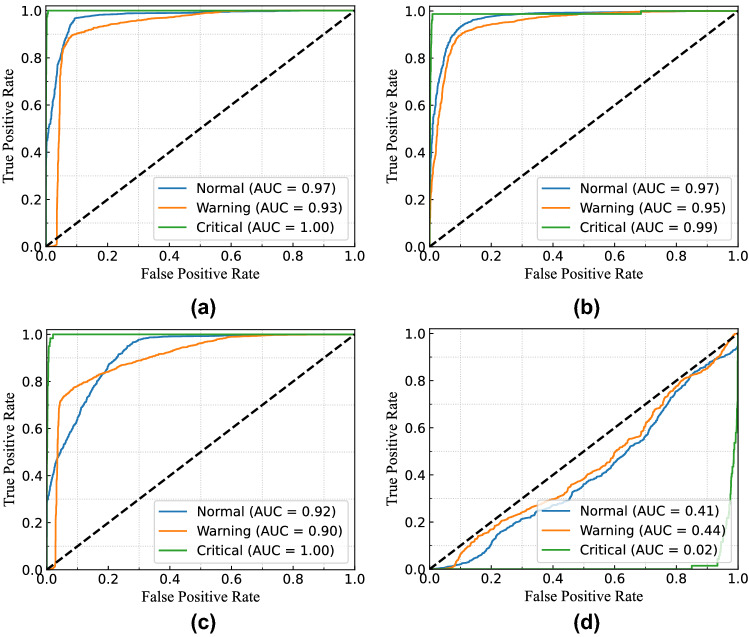
ROC curve of MSVM classifier for resistive leakage current with (**a**) linear kernel; (**b**) RBF kernel; (**c**) polynomial kernel; and (**d**) sigmoid kernel for $B_{1}$.

**Figure 21:**
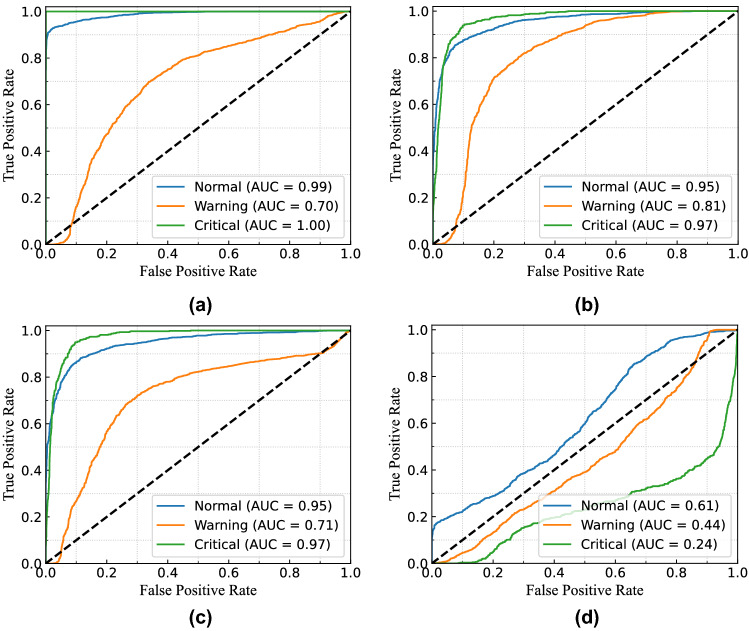
ROC curve of MSVM classifier for capacitive leakage current with (**a**) linear kernel; (**b**) RBF kernel; (**c**) polynomial kernel; and (**d**) sigmoid kernel for $B_{1}$.

For example, the system runs with the critical condition because of the leakage current, but the resistive leakage current is quite higher than the capacitive leakage current. In these cases, the possibility of resistive leakage current for the critical condition will be higher, whereas the capacitive leakage current will be responsible for normal or warning conditions. The probability density distribution of resistive and capacitive leakage currents will not be the same in this condition.

To execute the MSVM model, we have used an HP Z8 G4 Workstation with 256 Gb of Memory and Intel(R) Xeon(R) Gold 5222 CPU @ 3.80GHz 3.79 GHz processors. For calculating the computational time of a single MSVM model, a sample size of 2000 samples are used and 5.8786 seconds is spent on the whole execution. As a result, a single detection takes an average of 2.8816 milliseconds to compute. Since we have implemented three different models to detect abnormalities, the aggregated MSVM model takes 13.2159 seconds to complete the execution. Consequently, a single detection for the combined model takes an average of 6.6079 milliseconds to compute.

Therefore, for evaluating the performance of the proposed model, the receiver operating characteristic (ROC) curve for multi-class classification in three different cases is considered. Moreover, the area under the curve (AUC) summarizes the ROC curve that measures a model’s ability to differentiate among classes. The ROC curve consisted of true positive rate (TPR) and false positive rate (FPR) presenting the performance of the MSVM classification model at all classification thresholds. The TPR and FPR can be defined as follows:

$$\begin{aligned} TPR=\frac{TP}{(TP+FN)}, \\ FPR=\frac{FP}{(FP+TN)}, \end{aligned}$$

where $TP=$ number of true positives, $TN=$ number of true negatives, $FP=$ number of false positives, and $FN=$ number of false negatives. Furthermore, accuracy and F1-score (i.e. calculated from recall and precision) are two performance indices that are considered to evaluate the performance of the proposed MSVM. The mathematical formulation of accuracy and F1-score for the MSVM are expressed as follows:

$$\begin{aligned} Accuracy=\frac{(TP+TN)}{(TP+TN+FP+FN)}, \\ F1-Score=\frac{TP}{TP+.5(FP+FN)}. \end{aligned}$$

**Table 2:** Accuracy and F1-score of RBC-MSVM algorithm for detecting the fault.

Cases	KPI	MSVM-linear (%)	MSVM-RBF (%)	MSVM-poly (%)	MSVM-sigmoid (%)
Total	Accuracy	98.77	97.74	95.25	58.82
	F1-score	98.12	97.69	95.14	32.88
Resistive	Accuracy	92.89	89.88	82.3	47.03
	F1-score	90.11	87.98	81.41	31.52
Capacitive	Accuracy	94.56	93.38	87.65	37.89
	F1-score	95.75	93.57	88.52	32.42

**Figure 22:**
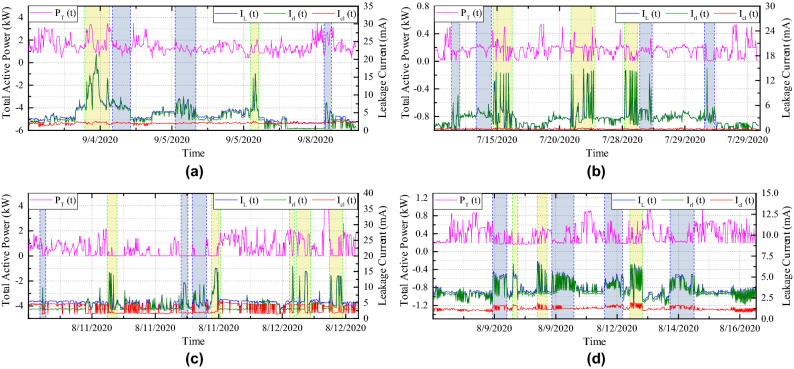
The monitoring samples results of (**a**) $B_{2}$, (**b**) $B_{3}$, (**c**) $B_{4}$, and (**d**) $B_{5}$.

To distinguish the performance of the RBC-MSVM classifier, we performed classification on varying kernel functions. Figure 19 illustrates the ROC curve and AUC of the MSVM model with different kernel functions in the case of total leakage current fault classification. The MSVM-RBF model has higher AUC, indicating that it is better at distinguishing between negative and positive classes, as seen in the figures. Furthermore, Table 2 shows the accuracy and F1-score of the implemented RBC-MSVM model. The results show that the MSVM-Linear, the MSVM-RBF, and the MSVM-Poly achieve higher accuracy and F1-score among the four MSVM models. Moreover, it also shows that the MSVM-Linear and MSVM-RBF achieve Accuracy = 98.77% and F1-score = 98.12% where MSVM-RBF has Accuracy = 97.74% and F1-score = 97.69%. The results demonstrate that the MSVM-Linear predicts all classified classes with less probability while MSVM-RBF has a greater probability. Despite the higher accuracy and F1-score of MSVM-Linear, the MSVM-RBF is highly convinced in its prediction. In addition, the MSVM-RBF and the MSVM-Poly also provide comparable results, with MSVM-RBF having a substantially better ROC curve than MSVM-Poly.

In the case of resistive leakage current fault classification, the ROC curve and the AUC results are presented in Fig. 20. According to the figures, the MSVM-Linear and the MSVM-RBF predict all classification classes with a comparable probability. And Table 2 shows both models possess 92.89% and 89.88 % accuracy, respectively, whereas F1-scores are 90.11% and 87.98 %. Consequently, the MSVM-RBF is more accurate in its prediction. Figure 21 presents the ROC curve and the AUC results for capacitive leakage current fault classification. The Table 2 shows that the MSVM-Linear outperforms the others in terms of accuracy and F1-score metrics. However, the value of AUC for MSVM-Linear in three classes are 0.99, 0.70, and, 1.00 while the MSVM-RBF has 0.95, 0.81, and 0.97. In the case of warning data classification, the MSVM-linear acquires a very poor AUC value than the MSVM-RBF. Consequently, the MSVM-linear show poor classification performance with low probability than the MSVM-RBF.

Following Fig. 22a–d depicts the variation of power and leakage current for the different periods received from the devices installed in separated buildings. We have also observed decomposed resistive and capacitive leakage current components to evaluate the effectiveness of the proposed MSVM classifier. Tables 3, 5, and 7 exhibit overall accuracy and F1 scores, while Tables 4, 6, and 8 provide AUC scores of RBC-MSVM based classifiers on household data.

**Table 3:** Accuracy and F1-score of RBC-MSVM algorithm for detecting the total leakage current fault.

Buil. No.	KPI	MSVM-linear (%)	MSVM-RBF (%)	MSVM-poly (%)	MSVM-sigmoid (%)
$B_{2}$	Accuracy	98.23	95.56	97.79	64.62
	F1-score	97.64	89.86	95.36	26.19
$B_{3}$	Accuracy	98.25	95.42	96.39	51.42
	F1-score	98.29	95.6	96.51	36.96
$B_{4}$	Accuracy	97.38	94.71	92.1	39.48
	F1-score	97.35	94.65	92.75	31.09
$B_{5}$	Accuracy	91.17	91.67	90.53	49.25
	F1-score	88.58	88.67	88.43	22.09

**Table 4:** AUC values of RBC-MSVM algorithm for detecting the total leakage current fault.

Buil. No.	Conditions	MSVM-linear	MSVM-RBF	MSVM-poly	MSVM-sigmoid
B_2_	Normal	1.00	1.00	1.00	0.88
	Warning	0.94	1.00	0.86	0.27
	Critical	1.00	1.00	1.00	0.09
B_3_	Normal	1.00	1.00	1.00	1.00
	Warning	0.81	0.95	0.96	0.59
	Critical	1.00	1.00	1.00	1.00
B4	Normal	0.99	0.96	0.97	0.66
	Warning	0.84	0.64	0.59	0.58
	Critical	1.00	1.00	1.00	1.00
B_5_	Normal	0.99	0.97	0.97	0.83
	Warning	0.88	0.88	0.88	0.64
	Critical	1.00	1.00	1.00	0.86

Overall, the accuracy of the MSVM-Linear models appear to be sufficient for most of the building, although the MSVM-RBF models have higher AUC values. In the scenario of leakage current fault classification, the highest accuracy and F1 score for $B_3$ are $98.25\%$ and $98.29\%$, respectively, with AUC scores of 1.00, 0.81,  and 1.00. Contrariwise, the MSVM-RBF models achieve comparable accuracy and F1 score with higher AUC values. Tables 3 and 4 show that the MSVM-Linear models outperform the others in terms of accuracy and F1 score, although the MSVM-RBF models detect all categorized classes more consistently. However, feature selection has evolved into a unique criterion for achieving acceptable accuracy. When the nature of the current changes, the accuracy of different kernel functions changes dramatically. During resistive leakage current fault classification, the MSVM-Linear classifier attained a maximum accuracy and F1 score of $96.15\%$ and $93.00 \%$ for $B_{2}$ with AUC scores of 1.00, 0.94,  and 1.00, whereas MSVM-RBF and MSVM-Poly demonstrated poorer performance metrics. By analyzing and comparing the results from Tables 5 and 6, the MSVM-Linear models perform better than others in terms of accuracy and F1 score, as well as the higher probability of detecting all categorized classes.

**Table 5:** Accuracy and F1-score of RBC-MSVM algorithm for detecting the resistive leakage current fault.

Buil. No.	KPI	MSVM-linear (%)	MSVM-RBF (%)	MSVM-poly (%)	MSVM-sigmoid (%)
$B_{2}$	Accuracy	96.15	95.92	94.15	67.62
	F1-score	93.00	92.24	90.10	26.89
$B_{3}$	Accuracy	94.12	92.38	82.27	65.86
	F1-score	92.18	90.25	74.91	48.77
$B_{4}$	Accuracy	92.94	91.31	86.50	30.11
	F1-score	92.06	90.53	86.16	30.76
$B_{5}$	Accuracy	91.85	91.45	90.30	49.00
	F1-score	89.75	89.31	88.36	21.92

**Table 6:** AUC values of RBC-MSVM algorithm for detecting the resistive leakage current fault.

Buil. No.	Conditions	MSVM-linear	MSVM-RBF	MSVM-poly	MSVM-sigmoid
B_2_	Normal	1.00	1.00	0.99	0.92
	Warning	0.93	0.92	0.94	0.66
	Critical	1.00	1.00	1.00	0.95
B_3_	Normal	1.00	0.99	1.00	1.00
	Warning	0.79	0.91	0.94	0.46
	Critical	1.00	1.00	1.00	1.00
B_4_	Normal	0.98	0.97	0.96	0.63
	Warning	0.71	0.70	0.72	0.60
	Critical	0.99	0.99	0.98	0.97
B_5_	Normal	1.00	0.96	0.95	0.85
	Warning	0.89	0.88	0.86	0.75
	Critical	1.00	1.00	1.00	0.87

**Table 7:** Accuracy and F1-score of RBC-MSVM algorithm for detecting the capacitive leakage current fault.

Buil. No.	KPI	MSVM-linear (%)	MSVM-RBF (%)	MSVM-poly (%)	MSVM-sigmoid (%)
$B_{2}$	Accuracy	86.08	88.17	84.92	54.00
	F1-score	79.67	86.11	76.08	52.3
$B_{3}$	Accuracy	89.75	91.94	86.71	49.79
	F1-score	73.29	89.98	84.95	49.95
$B_{4}$	Accuracy	93.94	95.5	84.94	48.06
	F1-score	86.64	88.4	84.53	41.24
$B_{5}$	Accuracy	86.6	92.15	85.6	38.38
	F1-score	85.26	88.84	83.13	35.28

**Table 8:** AUC values of RBC-MSVM algorithm for detecting the capacitive leakage current fault.

Buil. No.	Conditions	MSVM-linear	MSVM-RBF	MSVM-poly	MSVM-sigmoid
B_2_	Normal	0.98	0.98	0.98	0.50
	Warning	0.98	0.97	0.97	0.50
	Critical	1.00	1.00	0.98	0.79
B_3_	Normal	0.89	1.00	1.00	0.93
	Warning	0.92	0.94	0.86	0.84
	Critical	1.00	1.00	1.00	0.98
B_4_	Normal	0.99	0.99	0.99	0.72
	Warning	0.99	1.00	0.99	0.76
	Critical	0.99	0.96	0.99	0.21
B_5_	Normal	0.95	0.95	0.94	0.41
	Warning	0.94	0.94	0.93	0.41
	Critical	0.98	0.92	0.98	0.47

Furthermore, Tables 7 and 8 show the results for capacitive leakage current fault classification. The maximum accuracy and F1-scores for $B_{4}$, which belongs to the MSVM-RBF model, are 95.15% and 88.40%, respectively. The MSVM-RBF models had greater accuracy, F1 Score, and AUC values in most of the households, indicating that they are better at properly detecting and distinguishing between negative and positive classes, as shown in the Tables 7 and 8.

However, due to the nearly equal distribution of data into three clusters, the performance of the classifier for leakage current fault detection is significantly greater than other fault classification approaches. From the Tables, it can be observed that the performance of the Linear kernel outperforms other kernel functions in most cases of total leakage and resistive leakage current. The main underlying reason of Linear kernel outperforms is that the data nature is becoming more linear after the feature’s scaling. The total leakage and resistive leakage current data are more linear than capacitive leakage current in the case of some houses. As a consequence, the MSVM-RBF has been outperformed for capacitive current. Moreover, the volume of data sample of each category is also an important factor for achieving higher accuracy, F1-score, and AUC scores.

## Conclusion

This paper has presented a cloud-based electrical appliance’s health status monitoring system using LoRa connectivity. In this study, starting from designing the sensor until detecting the leakage current fault is elucidated. The scheme aims at developing a data-driven method to learn the permissible range of leakage current in finding the possible features by analyzing the relationship among different variables and detecting the fault by classifying the real-time data. The real-time data is successfully collected and stored in the cloud server through SMD and LoRa gateway. To assess the feasibility and performance of the proposed system, the RBC-MSVM based classification method is implemented on five buildings, yielding the highest accuracy ($98.23\%$) and the F1 score ($97.64\%$) when the system’s circumstances are appropriately distinguished. Furthermore, its fault detection capabilities and rapid detection time (on average 6.67 ms) suggest that it is commercially feasible. The MSVM classifier combined with the Linear/RBF kernel functions and RBC is a promising option for fault diagnosis of electrical safety monitoring equipment, based on the preceding results. In the future, the implementation of fault detection scheme on edge server will enable more accurate analysis of electrical appliance conditions and eliminates the sudden destructive incidents in the electrical system.

## Data Availability

The data that support the findings of this study are available from Information Technology Research Center (ITRC) but restrictions apply to the availability of these data, which were used under license for the current study, and so are not publicly available. Data are however available from the authors (Yeong Min Jang, email: yjang@kookmin.ac.kr ) upon reasonable request and with permission of ITRC.

## Functions

$$f(\cdot )$$: Decision function
$$k(\cdot )$$: Kernel function

## Parameters

$\delta _{I}$: Angle between terminal voltage and current
$\delta _{L}$: Angle between terminal voltage and leakage current
$\mathbf {N}$: Number of appliances
$$\theta$$: Weight vector
$$b$$: Biasing unit
$$C_{k}$$: $$k_{th}$$ cluster
$dt_{VI_{L}}$: XOR output ON-time for leakage current
$dt_{VI}$: XOR output ON-time for total entering current
$$f$$: Frequency
$$F_{k}$$: Scaled feature
$I_{cl}$: Capacitive leakage current
$I_{L,T}$: Total returning current
$I_{L}$: Total leakage current
$I_{rl}$: Resistive leakage current
$I_{T}$: Total enterning current
$$I_{Xc}$$: Capacitive current
$I_{Xlc}$: Sum inductive and capacitive current
$$I_{Xl}$$: Inductive current
$$I_{Zr}$$: Resistive current
$n$: Number of features
$$p.f.$$: Power factor
$P$: Total active power
$Q$: Total reactive power
$r$: Pearson correlation coefficient
$S_{T}$: Total apparent power
$$V_{LV}$$: Terminal voltage
$$x$$: Input vector
$$Z_{L}$$: Insulation impedence

## Sets, index and subscripts

$ap$: Index of appliances
$e$: Index of end
$i$: Index of data sample
$$in$$: Entering moment
$$j$$: Complex number
$k$: Index of cluster
$$ot$$: Returning moment
$s$: Index of start
$SoS$: Set of state of system
$t$: Index of time
$Th_{}^{C}$: Set of threshold for critical condition
$Th_{}^{N}$: Set of threshold for normal condition
$Th_{}^{W}$: Set of threshold for warning condition

## References

[1] Richard, C. *Home Electrical Fires* (National Fire Protection Association, 2019).

[2] Alavi AH, Jiao P, Buttlar WG, Lajnef N (2018). Internet of things-enabled smart cities: State-of-the-art and future trends. Measurement.

[3] Yu L, Li H, Feng X, Duan J (2016). Nonintrusive appliance load monitoring for smart homes: Recent advances and future issues. IEEE Instrum. Meas. Mag..

[4] Tong RT, Guo LZ, Cao Z (2013). The analysis in several application issues for leakage current electrical fire monitoring system. Procedia Eng..

[5] Jadhav, A. R., Kiran, S., M. P. R. & Pachamuthu, R. Development of a novel IoT-enabled power- monitoring architecture with real-time data visualization for use in domestic and industrial scenarios. In *IEEE Transactions on Instruments and Measurements*, vol. 70, 1–14 (2021).

[6] Peng C, Huang J (2016). A home energy monitoring and control system based on ZigBee technology. Int. J. Green Energy.

[7] Martani C, Lee D, Robinson P, Britter R, Ratti C (2012). ENERNET: Studying the dynamic relationship between building occupancy and energy consumption. Energy Build..

[8] ElShafee A, Hamed KA (2012). Design and implementation of a WiFi based home automation system. Int. J. Comput. Electr. Autom. Control Inf. Eng..

[9] Gan, S., Li, K., Wang, Y., & Cameron, C. IoT based energy consumption monitoring platform for industrial processes. In *Proceedings of UKACC 12th International Conference on Control (CONTROL), Sheffield* 236–240 (2018).

[10] Liu Q, Kamoto KM, Liu X, Sun M, Linge N (2019). Low-complexity non-intrusive load monitoring using unsupervised learning and generalized appliance models. IEEE Trans. Consum. Electron..

[11] Shafiei M, Golestaneh F, Ledwich G, Nourbakhsh G, Gooi HB, Arefi A (2020). Fault detection for low-voltage residential distribution systems with low-frequency measured data. IEEE Syst. J..

[12] Wu X, Han X, Liang KX (2019). Event-based non-intrusive load identification algorithm for residential loads combined with under determined decomposition and characteristic filtering. IET Gener. Transm. Distrib..

[13] Chen W, Gong Q, Geng G, Jiang Q (2020). Cloud-based non-intrusive leakage current detection for residential appliances. IEEE Trans. Power Deliv..

[14] Wang J, Xi Y, Fang C, Cai L, Wang J, Fan Y (2019). Leakage current response mechanism of insulator string with ambient humidity on days without rain. IEEE Access.

[15] Werneck MM, dos Santos DM, de Carvalho CC, de Nazaré FVB, da Silva Barros Allil RC (2015). Detection and monitoring of leakage currents in power transmission insulators. IEEE Sens. J..

[16] Harid N, Bogias AC, Griffiths H, Robson S, Haddad A (2016). A wireless system for monitoring leakage current in electrical substation equipment. IEEE Access.

[17] Shaikh MF, Park J, Lee SB (2021). A non-intrusive leakage flux based method for detecting rotor faults in the starting transient of salient pole synchronous motors. IEEE Trans. Energy Convers..

[18] Aoudi, W. Support vector machines: A distance-based approach to multi-class classification. In *Proceedings of IEEE International Multidisciplinary Conference on Engineering and Technology (IMCET)* 1–6 (2016).

[19] Gunn SR (2010). Support vector machines for classification and regression. Analyst.

[20] Samanta B (2003). Artificial neural network based fault diagnostics of rolling element bearings using time-domain features. Mech. Syst. Signal Process..

[21] Tian J, Morillo C, Azarian MH, Pecht M (2016). Motor bearing fault detection using spectral kurtosis-based feature extraction coupled with Knearest neighbor distance analysis. IEEE Trans. Ind. Electron..

[22] Yang Q, Ruan J, Zhuang Z, Huang D (2020). Fault identification for circuit breakers based on vibration measurements. IEEE Trans. Instrum. Meas..

[23] Liu S, Xu L, Li Q, Zhao X, Li D (2018). Fault diagnosis of water quality monitoring devices based on multiclass support vector machines and rule-based decision trees. IEEE Access.

[24] Gangsar, P., Ali, Z., Chouksey, M. & Parey, A. An intelligent and robust fault diagnostics for an electromechanical system using vibration and current signals. In *Recent Advances in Manufacturing, Automation, Design and Energy Technologies* 485–494 (Springer, 2022).

[25] Forouzesh A, Golsorkhi MS, Savaghebi M, Baharizadeh M (2021). Support vector machine based fault location identification in microgrids using interharmonic injection. Energies.

[26] Kazemi Z, Naseri F, Yazdi M, Farjah E (2021). An EKF-SVM machine learning-based approach for fault detection and classification in three-phase power transformers. IET Sci. Meas. Technol..

[27] Eslami M, Jannati M, Tabatabaei SS (2021). An improved protection strategy based on PCC-SVM algorithm for identification of high impedance arcing fault in smart microgrids in the presence of distributed generation. Measurement (Lond.).

[28] Behrends H, Millinger D, Weihs-Sedivy W, Javornik A, Roolfs G, Geißendörfer S (2022). Analysis of residual current flows in inverter based energy systems using machine learning approaches. Energies.

[29] Zhang, X., Wang, Y., Dou, Z., Wang, W., Bai, Y. Residual current fault type recognition based on S3VM and KNN cooperative training. *J. Power Electron.* (2022).

[30] Han X, Sheng W, Du S, Su J, Liu G (2017). Novel protection scheme for residual current device-based electric fault time detection and touch current identification. IET Gener. Transm. Distrib..

[31] LoRa. Accessed: Feb. 2021. [Online]. Available: https://lora.readthedocs.io/en/latest/#: :text=For%\$20uplink%2C%20the%20maximum%20transmission,day%20depending%20on%20the%20channel

[32] Rizzi M, Ferrari P, Flammini A, Sisinni E (2017). Evaluation of the IoT LoRaWAN solution for distributed measurement applications. IEEE Trans. Instrum. Meas..

[33] Alam B, Doja MN, Alam M, Mongia S (2013). 5-Layered architecture of cloud database management system. AASRI Procedia.

[34] Kofler, M. *What is MySQL: MySQL* (ed. Kofler, M.) 3–19 (Apress, 2001).

[35] Nasir IM, Khan MA, Yasmin M, Shah JH, Gabryel M, Scherer R, Damaševičius R (2020). Pearson correlation-based feature selection for document classification using balanced training. Sensors.

[36] Wand, Z. & Xue, X. Multi-class support vector machines. In *Support Vector Machines Applications* (eds. Ma, Y. & Guo, G.) 23–48 (Springer, 2014).

